# 2-(2-Fluoro-4-hy­droxy­benz­yl)isoindoline-1,3-dione

**DOI:** 10.1107/S1600536812029923

**Published:** 2012-07-18

**Authors:** Hilal Vesek, Canan Kazak, Ayşen Alaman Ağar, Sümeyye Gümüş, Muhittin Aygün

**Affiliations:** a Department of Physics, Faculty of Arts and Sciences, Ondokuz Mayıs University, Kurupelit, TR-55139 Samsun, Turkey; b Department of Chemistry, Faculty of Arts and Sciences, Ondokuz Mayıs University, Kurupelit, TR-55139 Samsun, Turkey; c Department of Physics, Faculty of Arts and Sciences, Dokuz Eylül University, Tınaztepe Kampüsü, TR-35160 Buca-İzmir, Turkey

## Abstract

In the title compound, C_15_H_10_FNO_3_, the dihedral angle between the isoindoline-1,3-dione and 3-fluoro-4-methyl­phenol groups is 86.88 (8)°. The isoindoline-1,3-dione fragment is almost planar, with an r.m.s. deviation of 0.0154 Å within the group. Inter­molecular C—H⋯O hydrogen bonds generate *C*(6) chains running parallel to the [010] direction.

## Related literature

For background to indoline-1,3-dione and its derivatives, see: Raza *et al.* (2010 ▶). For discussion of the broad spectrum of properties of these compounds, see: Bhattacharya & Chakrabarti (1998 ▶). For discussion of their anti-inflammatory properties, see: Sridhar & Ramesh (2001 ▶). For discussion of their anxiogenic activities, see: Medvedev *et al.* (1996 ▶). For related structures, see: Asad *et al.* (2012 ▶); Fu *et al.* (2010 ▶). For classification of hydrogen-bonding patterns, see: Bernstein *et al.* (1995 ▶).
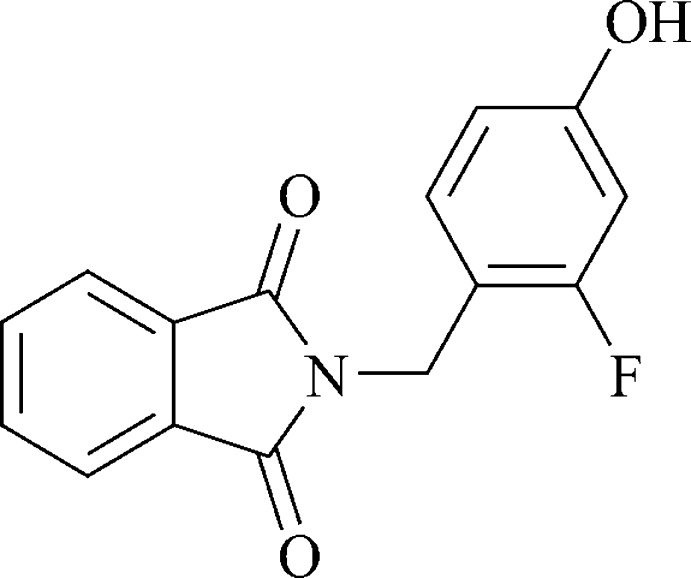


## Experimental

### 

#### Crystal data

C_15_H_10_FNO_3_
*M* *_r_* = 271.24
Monoclinic, 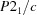

*a* = 12.4362 (7) Å
*b* = 13.8189 (8) Å
*c* = 7.2376 (4) Å
β = 105.784 (6)°
*V* = 1196.92 (12) Å^3^
*Z* = 4
Mo *K*α radiation
μ = 0.12 mm^−1^
*T* = 296 K
0.49 × 0.36 × 0.16 mm

#### Data collection

Agilent Xcalibur Eos diffractometer
Absorption correction: analytical [*CrysAlis PRO* (Agilent, 2012 ▶) and Clark & Reid (1995 ▶)] *T*_min_ = 0.977, *T*_max_ = 0.995
6558 measured reflections
2475 independent reflections
1455 reflections with *I* > 2σ(*I*)
*R*_int_ = 0.030

#### Refinement

*R*[*F* ^2^ > 2σ(*F* ^2^)] = 0.057
*wR*(*F* ^2^) = 0.141
*S* = 1.04
2475 reflections
182 parameters
1 restraint
H-atom parameters constrained
Δρ_max_ = 0.27 e Å^−3^
Δρ_min_ = −0.25 e Å^−3^

### 

Data collection: *CrysAlis PRO* (Agilent, 2012 ▶); cell refinement: *CrysAlis PRO*; data reduction: *CrysAlis PRO*; program(s) used to solve structure: *SHELXS97* (Sheldrick, 2008 ▶); program(s) used to refine structure: *SHELXL97* (Sheldrick, 2008 ▶); molecular graphics: *ORTEP-3 for Windows* (Farrugia, 1997 ▶); software used to prepare material for publication: *WinGX* (Farrugia, 1999 ▶).

## Supplementary Material

Crystal structure: contains datablock(s) I, global. DOI: 10.1107/S1600536812029923/mw2073sup1.cif

Structure factors: contains datablock(s) I. DOI: 10.1107/S1600536812029923/mw2073Isup2.hkl

Supplementary material file. DOI: 10.1107/S1600536812029923/mw2073Isup3.cml

Additional supplementary materials:  crystallographic information; 3D view; checkCIF report

## Figures and Tables

**Table 1:** Hydrogen-bond geometry (Å, °)

*D*—H⋯*A*	*D*—H	H⋯*A*	*D*⋯*A*	*D*—H⋯*A*
O1—H1⋯F1^i^	0.82	2.52	3.267 (2)	152
C2—H6⋯O2^ii^	0.93	2.51	3.303 (3)	144
C12—H12⋯O2^iii^	0.93	2.51	3.403 (3)	161
C15—H15⋯O3^iv^	0.93	2.47	3.346 (3)	157

Symmetry codes: (i) 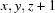; (ii) 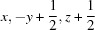; (iii) 
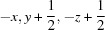
; (iv) 
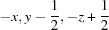
.

**Fig. 1.**
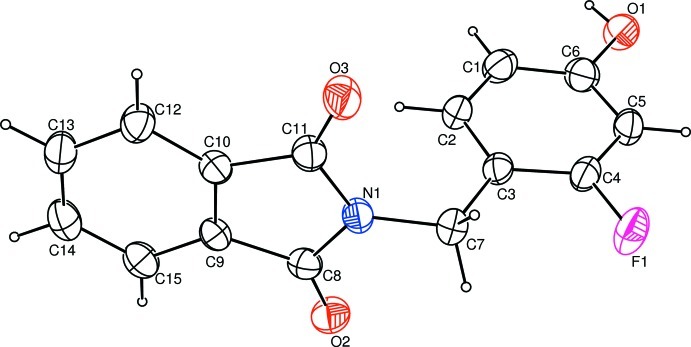
The molecular structure of the title compound, showing the atom-numbering scheme. Displacement ellipsoids are drawn at the 30% probability level.

**Fig. 2.**
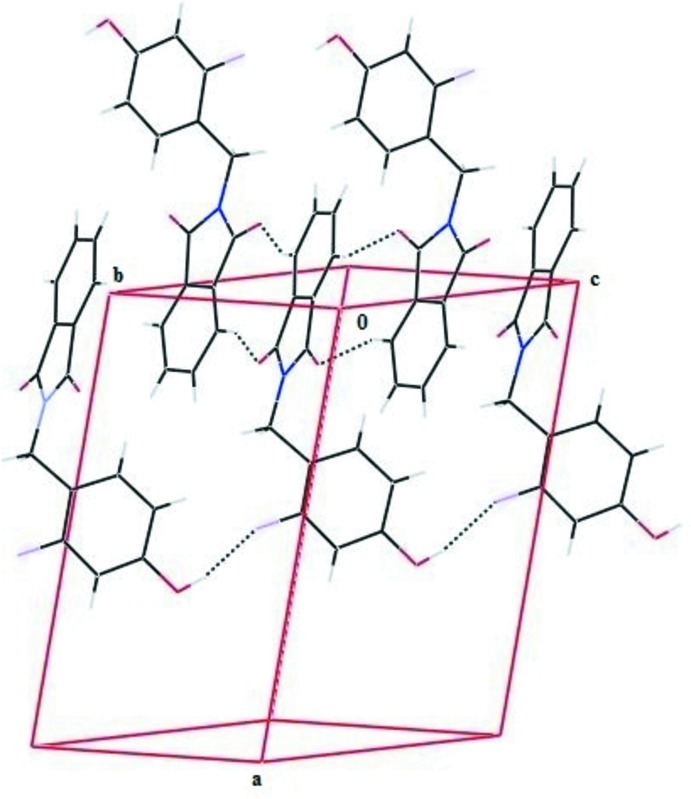
Crystal packing, viewed along the b axis,of the title compound. The C—H···O and O—H···F interactions are shown as dashed lines (see Table 1 for details).

## supplementary crystallographic information

### Comment

Indoline-2,3-dione and its derivatives are well known for their broad spectrum properties including anticonvulsant (Bhattacharya & Chakrabarti, 1998), anti-inflammatory (Sridhar & Ramesh, 2001) and anxiogenic (Medvedev *et al.*, 1996) activities. On the other hand, dithiocarbamates also show a large range of biological activities for example fungicidal (Ozkirimli *et al.*, 2005) and antitumor activities (Cao *et al.*, 2005; Gaspari *et al.*, 2006).

As an extension of the work on the structural characterization of indoline-2,3-dione derivatives, the crystal structure of the title compound is reported here. The isoindoline-1,3-dione fragment is almost planar with an r.m.s. deviation of 0.0154 Å within the group. This unit makes a dihedral angle of 86.88 (8)° with the benzene ring.

The F1—C4 bond length of 1.349 (3) Å agrees with the corresponding distance in 9-(7-fluoro-4-oxo-4*H*-chromen-3-yl)-3,3,6,6-tetramethyl-2,3,4,5,6, 7,8,9-octahydro-1*H*-xanthene-1,8-dione [1.349 (2) Å (Asad *et al.*, 2012)]. The C═O bond lengths are 1.205 (3) Å for C8═O2 and C11═O3 which are similar to the corresponding values found in 2-(2-oxothiolan-3-yl)isoindoline-1,3-dione [1.202 (5) Å and 1.207 (5) Å (Raza *et al.*, 2010)].

The molecules are linked into sheets by a combination of C—H···O and O—H···F interactions (Table 1). C(6) chains along [010] are created by pairwise C12—H12···O2 and C15—H15···O3 hydrogen bond interactions. The combination of the C(6) chains generates chain edge-fused *R*_2_^2^(10) rings running along [010]. C(6) chains along [001] are formed by O1—H1···F1 hydrogen bond interactions (Fig.2).

### Experimental

The compound 2-(2-fluoro-4-hydroxybenzyl)-1*H*-isoindole-1,3(2*H*)-dione was prepared by combining solutions of 2-hydroxy-1*H*-isoindole-1,3(2*H*)-dione (0.011 g 0.067 mmol) in 20 ml of ethanol and 1-(2,4-difluorophenyl)methanamine (0.009 g, 0.067 mmol) in 20 ml of ethanol and refluxing the resulting mixture for 1 h with stirring. Crystals of 2-(2-fluoro-4-hydroxybenzyl)-1*H*-isoindole-1,3(2*H*)-dione suitable for X-ray analysis were obtained from ethyl alcohol by slow evaporation (yield 72%; m.p. 155–158°C).

### Refinement

The H1 atom was located in a difference map and the O—H distance adjusted to 0.82 (2) Å while the other H atoms were placed in calculated positions. All were constrained to ride on their parent atoms, with C—H = 0.93–0.97 Å and *U*_iso_(H) = 1.2*U*_eq_(C,O).

### Crystal data

**Table:**

C_15_H_10_FNO_3_	*F*(000) = 560
*M**_r_* = 271.24	*D*_x_ = 1.505 Mg m^−^^3^
Monoclinic, *P*2_1_/*c*	Mo *K*α radiation, λ = 0.71073 Å
Hall symbol: -P2ybc	Cell parameters from 1089 reflections
*a* = 12.4362 (7) Å	θ = 3.3–29.3°
*b* = 13.8189 (8) Å	µ = 0.12 mm^−^^1^
*c* = 7.2376 (4) Å	*T* = 296 K
β = 105.784 (6)°	Plate, yellow
*V* = 1196.92 (12) Å^3^	0.49 × 0.36 × 0.16 mm
*Z* = 4	

### Data collection

**Table:**

Agilent Xcalibur Eos diffractometer	2475 independent reflections
Radiation source: Enhance (Mo) X-ray Source	1455 reflections with *I* > 2σ(*I*)
Graphite monochromator	*R*_int_ = 0.030
Detector resolution: 16.1333 pixels mm^-1^	θ_max_ = 26.5°, θ_min_ = 3.3°
ω scans	*h* = −15→15
Absorption correction: analytical [*CrysAlis PRO* (Agilent, 2012) and Clark & Reid (1995)]	*k* = −10→17
*T*_min_ = 0.977, *T*_max_ = 0.995	*l* = −9→9
6558 measured reflections	

### Refinement

**Table:**

Refinement on *F*^2^	Secondary atom site location: difference Fourier map
Least-squares matrix: full	Hydrogen site location: inferred from neighbouring sites
*R*[*F*^2^ > 2σ(*F*^2^)] = 0.057	H-atom parameters constrained
*wR*(*F*^2^) = 0.141	*w* = 1/[σ^2^(*F*_o_^2^) + (0.0576*P*)^2^ + 0.052*P*] where *P* = (*F*_o_^2^ + 2*F*_c_^2^)/3
*S* = 1.04	(Δ/σ)_max_ < 0.001
2475 reflections	Δρ_max_ = 0.27 e Å^−^^3^
182 parameters	Δρ_min_ = −0.25 e Å^−^^3^
1 restraint	Extinction correction: *SHELXL97* (Sheldrick, 2008), Fc^*^=kFc[1+0.001xFc^2^λ^3^/sin(2θ)]^-1/4^
Primary atom site location: structure-invariant direct methods	Extinction coefficient: 0.015 (2)

### Special details

**Table:**

Geometry. All e.s.d.'s (except the e.s.d. in the dihedral angle between two l.s. planes) are estimated using the full covariance matrix. The cell e.s.d.'s are taken into account individually in the estimation of e.s.d.'s in distances, angles and torsion angles; correlations between e.s.d.'s in cell parameters are only used when they are defined by crystal symmetry. An approximate (isotropic) treatment of cell e.s.d.'s is used for estimating e.s.d.'s involving l.s. planes.
Refinement. Refinement of *F*^2^ against ALL reflections. The weighted *R*-factor *wR* and goodness of fit *S* are based on *F*^2^, conventional *R*-factors *R* are based on *F*, with *F* set to zero for negative *F*^2^. The threshold expression of *F*^2^ > σ(*F*^2^) is used only for calculating *R*-factors(gt) *etc*. and is not relevant to the choice of reflections for refinement. *R*-factors based on *F*^2^ are statistically about twice as large as those based on *F*, and *R*-factors based on ALL data will be even larger.

### Fractional atomic coordinates and isotropic or equivalent isotropic displacement parameters (Å^2^)

**Table:**

	*x*	*y*	*z*	*U*_iso_*/*U*_eq_	
F1	0.51132 (13)	0.39770 (13)	0.2014 (2)	0.0744 (5)	
O1	0.62844 (14)	0.34620 (14)	0.8605 (2)	0.0669 (6)	
H1	0.6025	0.3378	0.9521	0.100*	
O2	0.14305 (15)	0.22946 (14)	0.1784 (3)	0.0688 (6)	
O3	0.15474 (15)	0.55595 (13)	0.2472 (2)	0.0640 (6)	
C1	0.4371 (2)	0.3540 (2)	0.7088 (4)	0.0587 (7)	
H2	0.4209	0.3437	0.8253	0.070*	
C2	0.3525 (2)	0.36576 (18)	0.5413 (4)	0.0512 (7)	
H6	0.2786	0.3635	0.5460	0.061*	
C3	0.37512 (19)	0.38080 (16)	0.3672 (3)	0.0432 (6)	
C4	0.4864 (2)	0.38324 (18)	0.3694 (3)	0.0489 (6)	
C5	0.5730 (2)	0.37230 (18)	0.5324 (4)	0.0555 (7)	
H3	0.6472	0.3745	0.5292	0.067*	
C6	0.5443 (2)	0.35796 (19)	0.6992 (4)	0.0573 (7)	
C7	0.2863 (2)	0.39461 (19)	0.1803 (3)	0.0513 (7)	
H7A	0.2988	0.4560	0.1244	0.062*	
H7B	0.2934	0.3438	0.0920	0.062*	
C8	0.1105 (2)	0.31010 (19)	0.1968 (4)	0.0486 (7)	
C9	0.00117 (19)	0.34221 (18)	0.2184 (3)	0.0443 (6)	
C10	0.00446 (19)	0.44188 (18)	0.2373 (3)	0.0423 (6)	
C11	0.1162 (2)	0.47537 (19)	0.2301 (3)	0.0464 (6)	
C12	−0.0859 (2)	0.4927 (2)	0.2606 (3)	0.0536 (7)	
H12	−0.0835	0.5596	0.2748	0.064*	
C13	−0.1802 (2)	0.4405 (2)	0.2620 (3)	0.0603 (8)	
H13	−0.2430	0.4731	0.2756	0.072*	
C14	−0.1837 (2)	0.3410 (2)	0.2438 (4)	0.0602 (8)	
H14	−0.2484	0.3079	0.2460	0.072*	
C15	−0.0921 (2)	0.2898 (2)	0.2221 (4)	0.0553 (7)	
H15	−0.0937	0.2228	0.2107	0.066*	
N1	0.17380 (16)	0.39322 (15)	0.2016 (3)	0.0475 (5)	

### Atomic displacement parameters (Å^2^)

**Table:**

	*U*^11^	*U*^22^	*U*^33^	*U*^12^	*U*^13^	*U*^23^
F1	0.0609 (10)	0.1071 (14)	0.0657 (9)	−0.0106 (9)	0.0350 (8)	−0.0019 (9)
O1	0.0467 (11)	0.0929 (15)	0.0528 (9)	−0.0076 (10)	−0.0006 (7)	0.0201 (10)
O2	0.0605 (13)	0.0456 (12)	0.1070 (15)	0.0039 (10)	0.0341 (11)	−0.0083 (11)
O3	0.0674 (13)	0.0437 (11)	0.0819 (13)	−0.0074 (10)	0.0220 (10)	−0.0014 (10)
C1	0.0578 (18)	0.0648 (18)	0.0557 (16)	−0.0048 (15)	0.0195 (13)	0.0081 (14)
C2	0.0432 (15)	0.0527 (16)	0.0620 (16)	−0.0025 (12)	0.0217 (12)	0.0039 (13)
C3	0.0406 (14)	0.0352 (13)	0.0560 (14)	−0.0028 (11)	0.0168 (11)	−0.0023 (11)
C4	0.0478 (16)	0.0486 (16)	0.0568 (15)	−0.0043 (13)	0.0253 (13)	−0.0021 (12)
C5	0.0404 (15)	0.0561 (18)	0.0729 (18)	−0.0019 (13)	0.0201 (14)	0.0016 (14)
C6	0.0463 (16)	0.0572 (17)	0.0596 (15)	−0.0028 (13)	−0.0008 (10)	0.0059 (14)
C7	0.0428 (14)	0.0555 (16)	0.0582 (14)	−0.0021 (13)	0.0182 (12)	0.0023 (13)
C8	0.0467 (16)	0.0416 (16)	0.0569 (15)	−0.0002 (13)	0.0132 (12)	−0.0039 (12)
C9	0.0415 (15)	0.0431 (15)	0.0461 (13)	0.0022 (12)	0.0084 (11)	−0.0006 (12)
C10	0.0400 (15)	0.0453 (15)	0.0389 (12)	0.0038 (12)	0.0061 (10)	−0.0012 (11)
C11	0.0489 (16)	0.0408 (16)	0.0465 (13)	−0.0005 (13)	0.0078 (11)	0.0014 (12)
C12	0.0541 (17)	0.0522 (16)	0.0524 (14)	0.0086 (14)	0.0108 (12)	−0.0037 (13)
C13	0.0474 (17)	0.075 (2)	0.0585 (15)	0.0138 (16)	0.0148 (13)	−0.0021 (15)
C14	0.0421 (16)	0.076 (2)	0.0631 (16)	−0.0033 (15)	0.0163 (13)	−0.0001 (15)
C15	0.0466 (16)	0.0528 (16)	0.0664 (16)	−0.0080 (14)	0.0153 (13)	−0.0047 (14)
N1	0.0387 (12)	0.0445 (12)	0.0589 (12)	0.0009 (10)	0.0126 (10)	−0.0005 (10)

### Geometric parameters (Å, º)

**Table:**

F1—C4	1.349 (3)	C7—H7A	0.9700
O1—C6	1.349 (3)	C7—H7B	0.9700
O1—H1	0.8200	C8—N1	1.388 (3)
O2—C8	1.205 (3)	C8—C9	1.478 (3)
O3—C11	1.205 (3)	C9—C15	1.374 (3)
C1—C6	1.354 (4)	C9—C10	1.384 (3)
C1—C2	1.382 (3)	C10—C12	1.373 (3)
C1—H2	0.9300	C10—C11	1.479 (3)
C2—C3	1.379 (3)	C11—N1	1.388 (3)
C2—H6	0.9300	C12—C13	1.380 (4)
C3—C4	1.380 (3)	C12—H12	0.9300
C3—C7	1.508 (3)	C13—C14	1.380 (4)
C4—C5	1.372 (3)	C13—H13	0.9300
C5—C6	1.364 (4)	C14—C15	1.385 (4)
C5—H3	0.9300	C14—H14	0.9300
C7—N1	1.449 (3)	C15—H15	0.9300
			
C6—O1—H1	109.5	O2—C8—C9	129.5 (2)
C6—C1—C2	118.4 (2)	N1—C8—C9	106.3 (2)
C6—C1—H2	120.8	C15—C9—C10	121.7 (2)
C2—C1—H2	120.8	C15—C9—C8	130.5 (2)
C3—C2—C1	121.5 (2)	C10—C9—C8	107.8 (2)
C3—C2—H6	119.2	C12—C10—C9	121.2 (2)
C1—C2—H6	119.2	C12—C10—C11	130.7 (2)
C2—C3—C4	116.5 (2)	C9—C10—C11	108.1 (2)
C2—C3—C7	123.9 (2)	O3—C11—N1	124.3 (2)
C4—C3—C7	119.6 (2)	O3—C11—C10	129.6 (2)
F1—C4—C5	118.2 (2)	N1—C11—C10	106.1 (2)
F1—C4—C3	118.0 (2)	C10—C12—C13	117.3 (3)
C5—C4—C3	123.9 (2)	C10—C12—H12	121.3
C6—C5—C4	116.3 (2)	C13—C12—H12	121.3
C6—C5—H3	121.8	C12—C13—C14	121.6 (3)
C4—C5—H3	121.8	C12—C13—H13	119.2
O1—C6—C1	119.6 (3)	C14—C13—H13	119.2
O1—C6—C5	117.1 (3)	C13—C14—C15	120.9 (3)
C1—C6—C5	123.3 (2)	C13—C14—H14	119.5
N1—C7—C3	113.3 (2)	C15—C14—H14	119.5
N1—C7—H7A	108.9	C9—C15—C14	117.2 (3)
C3—C7—H7A	108.9	C9—C15—H15	121.4
N1—C7—H7B	108.9	C14—C15—H15	121.4
C3—C7—H7B	108.9	C11—N1—C8	111.6 (2)
H7A—C7—H7B	107.7	C11—N1—C7	123.9 (2)
O2—C8—N1	124.2 (2)	C8—N1—C7	124.5 (2)
			
C6—C1—C2—C3	0.2 (4)	C8—C9—C10—C11	−0.5 (2)
C1—C2—C3—C4	0.2 (4)	C12—C10—C11—O3	1.8 (4)
C1—C2—C3—C7	−179.6 (2)	C9—C10—C11—O3	−177.4 (2)
C2—C3—C4—F1	179.9 (2)	C12—C10—C11—N1	−179.3 (2)
C7—C3—C4—F1	−0.3 (3)	C9—C10—C11—N1	1.6 (2)
C2—C3—C4—C5	−0.4 (4)	C9—C10—C12—C13	−0.7 (3)
C7—C3—C4—C5	179.4 (2)	C11—C10—C12—C13	−179.8 (2)
F1—C4—C5—C6	179.9 (2)	C10—C12—C13—C14	1.0 (3)
C3—C4—C5—C6	0.2 (4)	C12—C13—C14—C15	−0.4 (4)
C2—C1—C6—O1	−179.6 (2)	C10—C9—C15—C14	0.7 (3)
C2—C1—C6—C5	−0.4 (4)	C8—C9—C15—C14	−179.7 (2)
C4—C5—C6—O1	179.4 (2)	C13—C14—C15—C9	−0.4 (4)
C4—C5—C6—C1	0.2 (4)	O3—C11—N1—C8	177.0 (2)
C2—C3—C7—N1	1.6 (3)	C10—C11—N1—C8	−2.1 (2)
C4—C3—C7—N1	−178.2 (2)	O3—C11—N1—C7	−2.8 (4)
O2—C8—C9—C15	−0.5 (4)	C10—C11—N1—C7	178.11 (18)
N1—C8—C9—C15	179.6 (2)	O2—C8—N1—C11	−178.1 (2)
O2—C8—C9—C10	179.2 (3)	C9—C8—N1—C11	1.8 (3)
N1—C8—C9—C10	−0.7 (2)	O2—C8—N1—C7	1.7 (4)
C15—C9—C10—C12	−0.1 (3)	C9—C8—N1—C7	−178.4 (2)
C8—C9—C10—C12	−179.79 (19)	C3—C7—N1—C11	92.4 (3)
C15—C9—C10—C11	179.2 (2)	C3—C7—N1—C8	−87.4 (3)

### Hydrogen-bond geometry (Å, º)

**Table:**

*D*—H···*A*	*D*—H	H···*A*	*D*···*A*	*D*—H···*A*
O1—H1···F1^i^	0.82	2.52	3.267 (2)	152
C2—H6···O2^ii^	0.93	2.51	3.303 (3)	144
C12—H12···O2^iii^	0.93	2.51	3.403 (3)	161
C15—H15···O3^iv^	0.93	2.47	3.346 (3)	157

Symmetry codes: (i) *x*, *y*, *z*+1; (ii) *x*, −*y*+1/2, *z*+1/2; (iii) −*x*, *y*+1/2, −*z*+1/2; (iv) −*x*, *y*−1/2, −*z*+1/2.
